# The Ethical and Responsible Development and Application of Advanced Brain Machine Interfaces

**DOI:** 10.2196/16321

**Published:** 2019-10-31

**Authors:** Andrew David Maynard, Marissa Scragg

**Affiliations:** 1 School for the Future of Innovation in Society Arizona State University Tempe, AZ United States

**Keywords:** brain machine interface, ethics, neuroethics, bioethics, ethical innovation, responsible innovation, risk, risk innovation

## Abstract

Advanced brain machine interfaces provide potentially transformative approaches to treating neurological conditions and enhancing the performance of users. Yet, as technological capabilities continue to progress in leaps and bounds, there is a possibility that these capabilities outstrip our collective understanding of how to ensure brain machine interfaces are developed and used ethically and responsibly. In this case, there is an overt danger of rapid technological developments leading to unanticipated harm through a lack of foresight including threats to privacy, autonomy, self-identity, and other areas of personal and social value which, while hard to quantify, represent substantial risks. There is also a very real likelihood of such risks undermining value creation around the technologies and the associated enterprises, as key stakeholders push back against perceived and actual threats to what they, in turn, hold to be of value. In order to successfully traverse the resulting risk landscape, researchers and developers will need to become increasingly adept at integrating a sophisticated understanding of ethical and socially responsible innovation into their enterprises. Here, we illustrate how a “risk innovation” approach may provide novel insights into mapping out this landscape and revealing potentially blindsiding risks. We show how this approach can be used to illuminate challenges and opportunities to the successful, ethical, and responsible development of advanced brain machine interfaces. In addition, we emphasize how success will ultimately depend on the willingness of innovators and others to take ethical and responsible innovation seriously and to draw on the interdisciplinary and transdisciplinary expertise that is necessary to translate good intentions into positive outcomes.

## Introduction

Invasive technologies that enable machines to directly interface with regions of the brain have been the subject of research and development for some decades. The invention of cochlear implants in 1957 was a pivotal point in brain machine interface development [1], and since then, an increasingly sophisticated array of brain machine interface technologies have emerged, from deep brain stimulation techniques [2] to the high-density microelectrode arrays such as the Utah Array [3]. With intensifying interest in neurotechnologies reflected, in part, in large-scale research initiatives such as the US Brain Initiative and the Human Brain Project in Europe, brain machine interface technologies are continuing to attract considerable attention. However, recent technical advances published by Elon Musk and colleagues [4] suggest that a watershed in brain machine interface technology is approaching, which has the potential to not only revolutionize the treatment of neurological disorders but also transition brain machine interfaces from being firmly rooted in the domain of exclusive therapeutic devices to transformative and widely available performance-enhancing technologies.

As this technology continues to mature, there are questions associated with its ethical and responsible development and use that need to be addressed if the technology is to improve lives without causing unanticipated and potentially serious harm. Over the past several years, these have been explored extensively by ethicists, researchers, and others [5-9] and include a better understanding of the nature and acceptability of potential risks to health, behavior, and personality/sense of identity as well as issues including who benefits from the technology and whether access confers an unfair advantage on users. They also raise concerns associated with privacy, user autonomy—especially where users have limited control over implanted brain machine interfaces and the data they produce—and challenges of data and device security. On the other hand, the potential benefits of brain machine interfaces—both for therapeutic and enhancement uses—raise important ethical questions on the extent to which slowing or stopping development may disadvantage future beneficiaries of the technology. Most recently, the UK Royal Society grappled with these and similar questions in a comprehensive perspective on emerging brain machine interface technologies, concluding that “neural interface technologies will continue to raise profound ethical, political, social and commercial questions that should be addressed as soon as possible to create mechanisms to approve, regulate or control the technologies as they develop, as well as managing the impact they may have on society” [10].

Within this emerging landscape, developers of advanced brain machine interfaces are in urgent need of guidance on how to proceed appropriately if they are to ensure the value of the technology is fully realized, without overstepping ethical lines. However, few frameworks exist that explicitly aid researchers, businesses, and others in developing brain machine interface technologies that are ethical, responsible, and successful. One emerging framework that is potentially useful in revealing pathways forward around ethical and responsible development is that of “risk innovation” [11]. This is a framework we are in the process of developing and testing, which is designed to support pragmatic decisions around responsible and ethical innovation, within the often-tight constraints enterprises face as they push the bounds of what is possible. Here, we explore the applicability of a risk innovation approach to guiding the ethical and responsible development of advanced brain machine interfaces [12].

## Challenges of Developing New Technologies Ethically and Responsibly

Developers of potentially transformative technologies face an increasingly complex array of challenges as they strive to balance value creation and economic success with ethical and socially responsible development. Although they often have a vision of their technology being used to improve lives or the environment, they are frequently operating under conditions of extreme uncertainty, within tight resource constraints and with somewhat limited understanding of how their technology may affect others or be perceived by them. The result is a convoluted and shifting “risk landscape” that developers need to traverse in order to be successful and one that is heavily influenced by unconventional, indistinct, and often people- and society-oriented hurdles. Traversing this landscape depends on developing and applying a sophisticated understanding of stakeholder value and values and a commitment to protecting and supporting these, where appropriate. This is a metaphorical landscape that lies between good ideas and their successful development. It is littered with potential pitfalls and often includes obstacles that are outside the immediate expertise of those attempting to traverse it. As the coupling between technology innovation and society becomes increasingly tight, this landscape shifts more rapidly than it has in the past [13].

Although quantitative evidence for how this shifting landscape impacts innovation remains elusive, it is hard to miss the challenges that technology companies such as Facebook, Google, and Amazon are now facing because of their failure to map out and plan for potentially blindsiding risks including risks that often have their roots in societal expectations and norms. This is part of a broader trend where concerns associated with issues such as privacy, justice, and autonomy have an ever-larger impact on the challenges technology companies need to navigate in order to succeed. At least some of these challenges stem from businesses focusing on *shareholder* value while failing to recognize just how quickly disregarding *stakeholder* value (including value to customers, impacted communities, and publics more generally) can shut them down. This is a trend that companies developing genetically modified products have painfully learned to account for in recent years [14]. More broadly, these trends are indicative of the ever-more complex dynamic between technology innovation and society, which is increasing the unpredictability of the risk landscape in many cases.

In part, because of the evolving complexity of this risk landscape, there is a growing recognition that long-term value creation around technology innovations depends on ethical and socially responsible development and an ability to anticipate and take early action to avoid potential issues [15]. This is seen in approaches to, for example, anticipatory governance [16] and agile governance [17], which aim to equip businesses, regulators, and others with the insights, tools, and skills to anticipate and navigate potential challenges. It is also deeply embedded in thinking around responsible innovation [18], which strives to provide developers and others with a framework within which commercial success is tied to social responsibility.

These are all approaches that are directly relevant to the development of advanced brain machine interfaces. Advanced brain machine interfaces represent technological advances which, while potentially transformative in a positive way, are likely to face nontechnical hurdles that could derail them if there is no broad and sophisticated understanding of how to develop them ethically and responsibly. However, many approaches being explored are hard to translate into concrete actions, especially within a high-speed technology sector that is on the cutting edge of redefining what is possible.

## Risk Innovation

To address the broader challenges here, we are developing pragmatic approaches to innovating responsibly and successfully within the framework of “risk innovation.” This is an approach to innovation that recognizes that successful and responsible development of novel technologies cannot be predicated on treating future risks the same way as past risks and assuming that established risk assessment and management approaches will continue to be applicable. Rather, it encourages innovative ways of thinking about and acting on risk, which reveal novel pathways to successful and socially responsible technology innovation [11].

At the core of risk innovation is the concept of approaching risk as a threat to value, together with metaphorically visualizing risk as a landscape to be traversed, which lies between new ideas and their translation into successful products. Here, “value” is defined by the context surrounding a new technology or product and the stakeholders and communities potentially impacted by it. In this way, value may take on conventional attributes of health, wellbeing, environmental impact, security, and economic growth. However, it may also take on equally important but often less tangible—and frequently overlooked—attributes such as dignity, identity, justice, privacy, and autonomy.

This framing of risk enables innovators and others to consider the question “risk to whom or what?” and not simply to the immediate enterprise. Within a tightly coupled world, threats to what is of value to investors, customers, and communities become threats to the enterprise or technology. For instance, a technology that threatens the privacy of key communities (or is perceived to do so) has the potential to elicit a backlash which, in turn, threatens trust in the technology and its ultimate adoption. Likewise, corporate decisions that threaten what is important to the workforce—for instance, developing military applications without transparency or consultation—has the potential to threaten the ability of a company to retain skilled employees [19].

In our work, we are developing tools and approaches that use the ideas behind risk innovation to enable innovators to better understand and navigate the risk landscape they face. Our methodology is designed to foster a risk innovation mindset. It is grounded in helping innovators first identify key areas of value to their organization, their investors, their consumers, and their communities and then identify and address “orphan risks” [20] that have the potential to threaten these areas of value. These are risks that are easy to overlook and ignore and yet have the ability to blindside development if they are not mapped out and addressed.

Within this approach, we focus on 18 specific orphan risks that span three domains covering social and ethical factors, organizations and systems, and unintended consequences of emerging technologies (Textbox 1). This is not an exhaustive list of orphan risks, and with one or two exceptions, it does not include more conventional risks for which there are established risk assessment and management tools and approaches. For example, cyber security is not explicitly listed, although less-obvious dimensions of cyber-based risks such as privacy and loss of agency are listed. Rather, these orphan risks were identified through assessment of entrepreneurial risk landscapes as being important to raising awareness around often-overlooked and hard-to-quantify risks. They are also intended to stimulate innovative approaches to identifying and navigating similar risks that do not appear on the list.

By starting with areas of value and then considering relevant orphan risks, this approach provides innovators with unique insights into potential pitfalls and steps that may be taken to navigate around them. It is an approach that is aimed at supporting successful development, while, at the same time, avoiding threats to value amongst communities that, in turn, have the ability to stymie progress. In doing so, it actively encourages ethical, responsible, and successful development. Importantly, our approach is explicitly designed to help innovators and others avoid the dangers of ignoring unfamiliar risks or paralysis by analysis as they grapple with being overwhelmed by a deluge of speculative risks.

**Orphan risks used within our “risk innovation” methodology that have the potential to undermine value creation in unexpected ways if not mapped out and addressed.**

**Organizations and systems**

- Bad actors: Risks from enterprises that behave in ways that are ethically questionable or that lead to unacceptable harm.
- Geopolitics: Risks from a lack of awareness of or strategies for navigating a shifting geopolitical landscape.
- Governance and Regulation: Risks from often evolving laws, policies, and practices that govern and guide business operations.
- Organizational Values and Culture: Risks from tensions between business practices, both internal and external, and the set of values that reflect what is important to a business’ founders and members.
- Reputation and Trust: Risks from a business that has only a rudimentary understanding of how their behavior and actions strengthen or weaken reputation and trust.
- Standards: Risks from a business’ lack of engagement with an evolving operational framework for businesses that spans legal requirements, informal guidelines, and norms and codes.

**Unintended consequences of emerging technologies**

- Black Swan Events: Risks from very-low-probability but high-impact events.
- Co-opted Tech: Risks from technologies and products that are used in ways that undermine the intention of the original business or business owner.
- Heath and Environment: Risks from new technologies and the products they are associated with, behaving in sufficiently novel ways that they potentially lead to threats to human health and the environment.
- Intergenerational Impacts: Risks from technologies that have potential impacts from one generation to another.
- Loss of Agency: Risks from products or business practices that reduce the ability of organizations and individuals to have agency.
- Product Lifecycle: Risks from unintended impacts of where and how a product’s materials are sourced and manufactured, how it is used, and its disposal and reuse.

**Social and ethical factors**

- Ethics: Risks from business practices overstepping the often-indistinct line between ethical and unethical behavior.
- Perception: Risks created from how people perceive a technology to impact/threaten what they think is important.
- Privacy: Risks from the social pitfalls associated with the use and misuse of an individual’s data.
- Social Justice and Equity: Risks from business practices and technologies that marginalize or disadvantage specific segments within society.
- Social Trends: Risks from shifts in social norms, changing consumer expectations, or evolving cultural behaviors.
- Worldview: Risks from people’s deeply held beliefs about how they view the world and how it should function.

## Applying a Risk Innovation Approach to Advanced Brain Machine Interfaces

The risk innovation framework we are developing is particularly pertinent to the advanced brain machine interface technology outlined by Musk et al [4]. Although the technological advances presented do not explicitly address the pathway between these developments and their successful application, a risk innovation approach provides insights into where there are potential threats to value—or development pinch-points—that may arise, and early actions that may be taken to navigate these. Importantly, it opens the way to win-win scenarios between technological functionality, commercial success, and ethical and socially responsible innovation.

Based on the technology described, we explore below how risk innovation may be used to provide a starting point for thinking through the potential challenges and opportunities surrounding the ethical and socially responsible development of the technology. This application is necessarily limited by assumptions around the development and use of the technology. However, it nevertheless provides novel insights into the technology’s development and enables questions associated with ethical, responsible, and successful innovation to be bound in ways that potentially reveal useful pathways forward.

As Musk et al [4] articulated, there is clear value in the advanced brain machine interface systems they describe for therapeutic use. This is a technology that has the potential to provide novel approaches for addressing a spectrum of neurological conditions, from Parkinson disease and epilepsy to debilitating migraines. It could also vastly improve how users are able to interface with and control prosthetics, including those that replace diminished sensory function.

There is also the expectation of the technology being used to enhance performance in healthy users. Although this is not articulated in the paper, Elon Musk and Neuralink have been explicit about the possibilities of using technology to augment the capabilities of users through high-speed, wireless brain machine interfaces [21]. Here, apps could be as diverse as aimed at gaming and cognitive enhancement or sensory enrichment.

These potential capabilities translate into the hypothesized (and not exclusive) areas of value for the enterprise, investors, customers, and communities that are listed in Textbox 2. These are indicative only, but illustrate how areas of value potentially vary between different groups. For instance, investors are likely to be interested in how others perceive the trustworthiness of developers as a proxy for long-term return on investment. Customers are likely to value the degree to which brain machine interfaces are secure from interference and ensure that they, as users, retain autonomy over their actions and brain function. On the other hand, communities potentially touched by the technology are likely to place a high premium on people with brain machine interfaces becoming somehow “other” and a threat to social norms and expectations.

Comparing the areas of value in Textbox 2 and the orphan risks listed in Textbox 1, it is possible to map relevant risks onto key areas of value. This exercise is necessarily subjective. However, it begins to provide novel insights into potential challenges worth exploring further if key areas of value are to be protected and nurtured.

**Figure 1:**
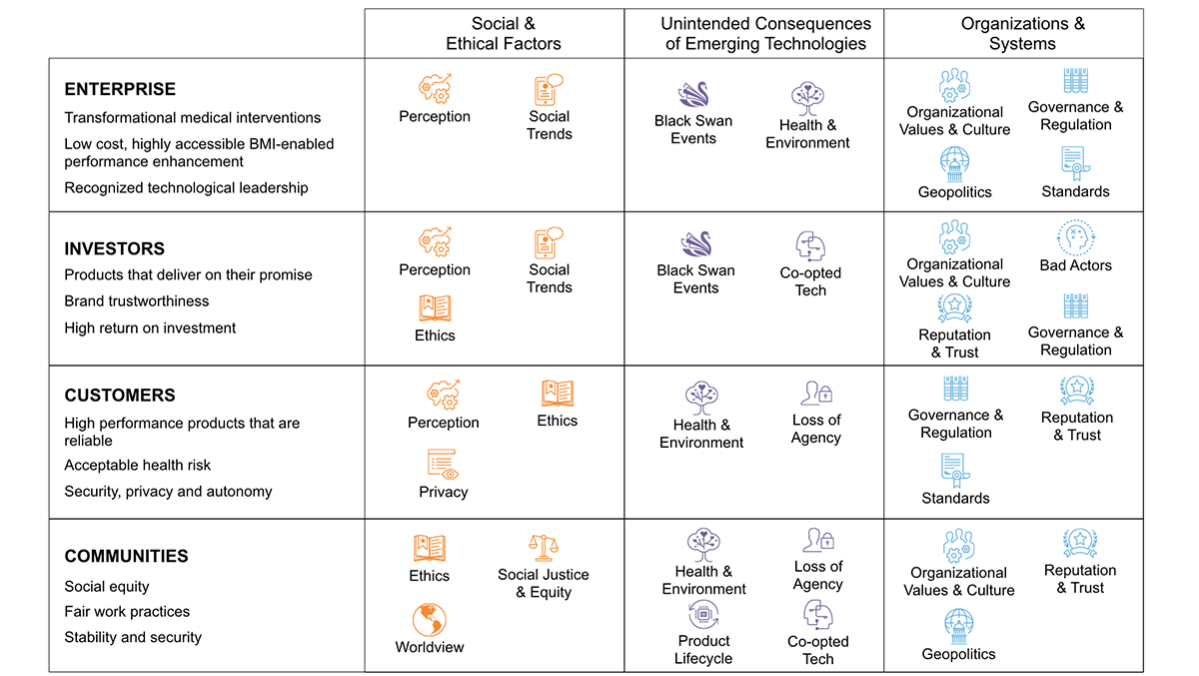
A qualitative snapshot of the possible orphan risk landscape associated with advanced brain machine interfaces.

Figure 1 provides an example of what this metaphorical “risk landscape” might look like for the brain machine interface, as described by Musk et al [4]. The approach is designed to provide a snapshot of areas that warrant further attention and to illuminate potential risk clusters that may otherwise remain obscured. It is not inclusive or comprehensive and does not include many conventional risks for which there are established risk assessment and mitigation frameworks, for example, cyber security. Rather, it provides a starting point for mapping out orphan risks that have the potential to directly or indirectly affect development and that could raise substantial issues around ethical and socially responsible innovation.

Here, it should be emphasized that this mapping exercise is qualitative and designed to focus and constrain perspectives on potentially blindsiding risks while encouraging innovative thinking around potential barriers to success. The methodology is intended to be iterative and open up new possibilities, without being prescriptive.

Within this context, the mapping presented in Figure 1 can be interpreted in a number of ways. First, it provides insights into orphan risks that are worth the enterprise being aware of. In the case of advanced brain machine interfaces, how investors, users, and others perceive the potential workings and impacts of the technology are flagged as important, as are social trends that may either create opportunities for development or potential barriers—for instance, if there is a public backlash against brain machine interface-based augmentation. Perhaps, not surprisingly, for an invasive neural read-write technology, novel health impacts are flagged, as are fundamentally unpredictable “black swan” events. In this case, the mapping suggests a high level of attention should be paid to enterprise and technology agility with respect to navigating around unexpected hurdles.

**Hypothesized areas of value associated with advanced brain machine interfaces. These are used as an example of how a risk innovation approach can help map out and navigate a complex risk landscape. They do not necessarily reflect areas of value as defined by the developers of advanced brain machine interfaces. The textbox is intentionally limited to three areas of value per column in order to avoid paralysis by overanalysis.**

**Enterprise**

- Transformational medical interventions
- Low cost, highly accessible brain machine interface–enabled performance enhancement
- Technological leadership

**Investors**

- Products that deliver on their promise
- Brand trustworthiness
- High return on investment

**Consumers**

- High performance products that are reliable
- Acceptable health risk
- Security, privacy, and autonomy

**Communities**

- Social equity
- Fair work practices
- Stability and security

Reading across the map, there is a greater density of orphan risks identified under “Organizations and Systems,” suggesting that some of the greatest potential threats to successful advanced brain machine interface technologies lie within the enterprise and the formal organizations governing how it operates. These include the degree to which organizational values and culture align with how the technology is developed and used, evolving regulations that may play an outsized influence on development pathways, and national and international standards that guide and limit technology performance and use.

Second, the risk landscape in Figure 1 provides insights into potential threats to value within key constituencies, which may, in turn, lead to barriers to success. Here, orphan risks that do not directly impact the enterprise, but are likely to influence its success, are highlighted. These include the ethics surrounding how the technology is developed and used, how it potentially leads to social injustice, and whether it raises privacy concerns. This broader perspective on orphan risks also flags possible issues such as how trustworthy the enterprise is perceived to be and the dangers of “bad actors” giving the technology a negative reputation that risks poisoning the market.

A third way the landscape in Figure 1 can be read is by identifying risks that cluster and converge in ways that increase the chances of truly blindsiding impacts. Certain risks dominate the landscape, including those associated with ethics, health, governance, and regulation. If not planned for, these could build upon each other to create insurmountable obstacles to value creation. Instead of planning for just one risk at a time, analysis and understanding of these clusters have the potential to provide insights into where resources are best focused to protect stakeholder value. Here, the analysis suggests that some of the more substantial near-time challenges to ensuring successful, responsible, and ethical advanced brain machine interfaces are associated with organizational systems and operations. These include internal processes such as the development and application of guiding principles and expectations of ethical and responsible behavior. However, they also extend to professional bodies, regulatory agencies, and communities of practice that form the broader ecosystem within which advanced brain machine interfaces are conceived, developed, and used. Here, Figure 1 also highlights orphan risks that occur multiple times such as ethics, perception, health and environmental impacts, and reputation and trust. These (and similar risk clusters) provide insights into where particular attention is most likely needed to support successful and responsible development.

## Summary

Applying a risk innovation methodology to the brain machine interface technology described by Musk et al [4] is admittedly subjective, especially as their paper primarily focuses on technological advances, and not on how they may be fully implemented into products and services in the future. However, its application provides a unique framework for exploring the ethical and socially responsible development of the technology in ways that are bounded by pragmatic considerations and are neither misguided by myopic optimism nor stymied by overspeculative pessimism. The approach taken here is intentionally focused on identifying pathways to innovation success and can thus be seen to favor the enterprise. Yet, by broadening the understanding of risk to key constituencies that are potentially impacted by the enterprise and the technology, it provides a canvas on which successful innovation may be aligned with ethical and socially responsible innovation. We would go so far as to say that it provides a conceptual framework and methodology that enables innovators to create and grow value by being intentionally responsive to ethical and social issues.

In the case of the brain machine interface technology described by Musk et al [4], this approach indicates that there is a crowded risk landscape between the current state of the technology and its successful development and use, with many of these risks pivoting on how companies address potentially serious ethical and social issues. These include the potential for the technology to widen the gap between the rich and poor (and the privileged and the marginalized); to challenge social norms around the use of enhancement technologies; and to raise complex issues around data privacy, user security, and autonomy—especially where manufacturers retain ownership of implanted brain machine interfaces or their operation and upkeep is dependent on a subscription service for instance. They also highlight the need for developers and others to consider with some seriousness how they build trust with the communities they depend on and actions that may erode trust.

By mapping out the orphan risk landscape they face—and iterating frequently as the landscape shifts and changes—there is no reason why the technical developments beginning to emerge around advanced brain machine interfaces will not lead to powerful therapeutic interventions and even transformative enhancement capabilities, which are economically successful, ethical, and socially responsible. Yet, this will only come about if developers are capable of looking beyond technical performance and conventional risks, culturing a sophisticated understanding of the risk landscape they face and the approaches they need to adopt in order to successfully navigate it.

The risk innovation approach we have described provides novel insights into this landscape and is a useful tool for revealing potentially blindsiding risks while there is still time to take corrective action. In the case of advanced brain machine interfaces, it helps map out challenges and opportunities that may otherwise be easy to miss, but have the ability to derail progress. Yet, it is just the start of a journey toward developing products that are capable of changing lives for the better, without causing substantial harm. Here, success depends on the willingness of innovators and others to take ethical and responsible innovation seriously and to draw on the interdisciplinary and transdisciplinary expertise that is necessary to translate good intentions into positive outcomes. Here, our hope is that this is a pathway that advanced brain machine interface technologies will follow.
